# A phylogenomic study of Iridaceae Juss. based on complete plastid genome sequences

**DOI:** 10.3389/fpls.2023.1066708

**Published:** 2023-01-31

**Authors:** Kashish Kamra, Joonhyung Jung, Joo-Hwan Kim

**Affiliations:** Department of Life Sciences, Gachon University, Seongnam, Gyeonggi, Republic of Korea

**Keywords:** plastid genome, Iridaceae, subfamilial classification, comparative genomics, phylogenetic analyses, genomic events

## Abstract

The plastid genome has proven to be an effective tool for examining deep correlations in plant phylogenetics, owing to its highly conserved structure, uniparental inheritance, and limited variation in evolutionary rates. Iridaceae, comprising more than 2,000 species, includes numerous economically significant taxa that are frequently utilized in food industries and medicines and for ornamental and horticulture purposes. Molecular studies on chloroplast DNA have confirmed the position of this family in the order Asparagales with non-asparagoids. The current subfamilial classification of Iridaceae recognizes seven subfamilies—Isophysioideae, Nivenioideae, Iridoideae, Crocoideae, Geosiridaceae, Aristeoideae, and Patersonioideae—which are supported by limited plastid DNA regions. To date, no comparative phylogenomic studies have been conducted on the family Iridaceae. We assembled and annotated (*de novo*) the plastid genomes of 24 taxa together with seven published species representing all the seven subfamilies of Iridaceae and performed comparative genomics using the Illumina MiSeq platform. The plastomes of the autotrophic Iridaceae represent 79 protein-coding, 30 tRNA, and four rRNA genes, with lengths ranging from 150,062 to 164,622 bp. The phylogenetic analysis of the plastome sequences based on maximum parsimony, maximum likelihood, and Bayesian inference analyses suggested that *Watsonia* and *Gladiolus* were closely related, supported by strong support values, which differed considerably from recent phylogenetic studies. In addition, we identified genomic events, such as sequence inversions, deletions, mutations, and pseudogenization, in some species. Furthermore, the largest nucleotide variability was found in the seven plastome regions, which can be used in future phylogenetic studies. Notably, three subfamilies—Crocoideae, Nivenioideae, and Aristeoideae—shared a common *ycf*2 gene locus deletion. Our study is a preliminary report of a comparative study of the complete plastid genomes of 7/7 subfamilies and 9/10 tribes, elucidating the structural characteristics and shedding light on plastome evolution and phylogenetic relationships within Iridaceae. Additionally, further research is required to update the relative position of *Watsonia* within the tribal classification of the subfamily Crocoideae.

## 1 Introduction

Iridaceae are one of the largest families of flowering plants within the order Asparagales, contains approximately 2,244 species in 66 genera worldwide, and is extensively diverse in southern Africa (Angiosperm Phylogeny Group et al., 2016; Christenhusz and Byng, 2016). Iridaceae comprises typically perennial evergreen or deciduous herbs, sometimes annuals (*Sisyrinchium* spp.), rarely shrubs with remarkable secondary growth, such as *Nivenia*, *Witsenia*, and *Klattia*, or mycoheterotrophic and achlorophyllous (*Geosiris*) (Goldblatt et al., 1998). Rootstock is a rhizome, corm, bulb, or woody caudex (shrub). Iridaceae can be distinguished from related lower asparagoid families by some distinguishable features, such as three-staminode flowers, styloid crystals, and isobilateral unifacial leaves (Rudall et al., 2003). The genera *Iris*, *Gladiolus*, *Freesia*, and *Crocus* are commonly used as ornamental cultivars, whereas corms of various Iridaceae species (*Moraea*, *Gladiolus*, *Ixia*, *Watsonia*, and *Babiana*) are eaten raw by indigenous people (Metelerkamp and Sealy, 1983). The spice saffron is prepared from the dried stigmas of *Crocus sativus*. Additionally, flowers are zygomorphic or actinomorphic, bisexual, sessile or pedicellate, epigynous, sometimes hypogynous (*Isophysis*), and with three stamen (rarely two) (*Diplarrena*). The flowering unit (inflorescence) is a terminal spikelet, spike, or panicle of a cluster of one–many monochasial rhipidia, individual flower, underground in *Geosiris* (Goldblatt et al., 1998).

Prior to the molecular approach, the association of Iridaceae within the order Asparagales was not widely accepted solely based on morphology (Dahlgren and Bremer, 1985). Recent molecular analyses of monocotyledons have consistently defined Iridaceae in the order Asparagales, among the non-core asparagoid grade, associated with families such as Doryanthaceae, Ixilirionaceae, and Tecophiliaceae (Kim et al., 2010). Goldblatt significantly discussed the morphological characteristics of Iridaceae and assumed that the putative inherited characteristics support a place in the Asparagales rather than Liliales (Goldblatt et al., 1998; Tillich, 2003). In addition, several molecular studies have undergone revised subfamilial circumscriptions and have been classified by various authors (Dahlgren and Bremer, 1985; Goldblatt, 1990; Rudall, 1994; Rudall, 1995; Souza-Chies et al., 1997; Reeves et al., 2001). The most updated phylogenetic reconstruction of Iridaceae is that of Goldblatt, using molecular data derived from five coding regions (*rbc*L, *rps*4, *trn*L-F, *mat*K, and *rps*16), validating the majority of classification aspects and recognizing seven subfamilies within Iridaceae (Isophysidoideae consisting of the monospecific *Isophysis tasmanica*, solely with superior ovary, Nivenioideae, Iridoideae, Crocoideae, Geosiridoideae, Aristeoideae, and Patersonioideae) (Goldblatt et al., 2008). Although these studies provide insights into the molecular systematics of Iridaceae and employ limited DNA regions, comprehensive phylogenetic studies with plastid genome sequences have not yet been conducted.

Chloroplasts are a significant semi-autonomous plant cell organelle with unique genetic information. Plastid (cp) genomes are maternally, paternally, or biparentally inherited within the angiosperm lineage (Birky Jr, 1995). In recent years, the plastid genome database has provided new opportunities for determining the evolutionary and phylogenetic relationships among plants because of their unique characteristics, such as small size, highly conserved gene content and order, and low recombination rates. Plasmid genomes have been used to investigate evolution and phylogeny. Typically, angiosperms have highly conserved cp or plastid genomes that are circularly organized and contain a large single-copy region (LSC) and a small single-copy region (SSC) separated by paired inverted repeats (IRs). In general, the cp genomes of terrestrial plant species include 120–130 distinct genes, the majority of which encode proteins associated with photosynthesis (approximately 79), with the remaining genes encoding transfer RNAs (tRNAs) (approximately 30) and ribosomal RNAs (rRNA) (4) (Dyer, 1984; Sugiura, 1992). The rapidly increasing accessibility of characterized plastomes determined by cost-effective next-generation sequencing (NGS) can help elucidate our understanding and facilitate insights into chloroplast gene function and evolution.

Currently, the genome sequences of some genera of the subfamily Iridoideae (*Iris* spp.), Geosiridoideae (*Geosiris australiensis* and *Geosiris aphylla*), and Crocoideae (*Crocus* spp.) are openly accessible in the National Center for Biotechnology Information (NCBI) (Joyce et al., 2018; Nemati et al., 2019; Kang et al., 2020). Furthermore, independent mycoheterotrophic and non-photosynthetic *Geosiris* species have had significant gene losses (Joyce et al., 2018). Therefore, more information should be acquired regarding the plastid genome resources of Iridaceae species. A comparative examination of the plastome sequences of various species can help investigate sequence variability and molecular evolutionary processes related to gene duplication, transfer, and rearrangement measures (Walker et al., 2014; Yan et al., 2018). We annotated and compared 24 newly sequenced representative taxa to understand the plastome evolution of Iridaceae in terms of their structure, gene contents and rearrangements, and IR expansion and contraction. Based on new genomic data and already available genomes in NCBI, we performed the first comprehensive phylogenomic analysis representing all seven subfamilies and tribes, except *Tritoniopsis*, to identify molecular signatures comprising insertions and deletions (INDELS), gene losses, and pseudogenization in DNA sequences that are uniquely shared by different species within Iridaceae. In this study, the cp genome sequence was obtained, and its gene content and organization were assessed. The second step involved a comparative analysis of several Iridaceae species. Finally, high nucleotide diversity allowed for the identification of genes that were extremely variable and informative. The plastid genome sequences can help reveal the evolutionary and phylogenetic relationships among the seven subfamilies and tribes of Iridaceae.

## 2 Materials and methods

### 2.1 Taxon sampling and DNA extraction

Fresh leaves of the representative taxa were collected and dried in silica gel until DNA extraction (**Table 1**). For the ingroup dataset, a total of 31 species, including 24 novel plastid genomes representing all seven Iridaceae subfamilies, were selected for analysis. Eleven outgroup species were used for comparison. The modified cetyl trimethyl ammonium bromide method was used to isolate total DNA (Doyle and Doyle, 1987) from the silica-dried samples. We also extracted the total cellular DNA from the three closely related Asparagales families (listed in **Table 1**) to choose the ideal set of outgroup species to construct phylogenomics. The extracted DNA concentration was measured using a Biospec-nano (Shimadzu) Spectrophotometer and tested using 1% agarose gel electrophoresis.

**Table 1:** List of newly sequenced species used in this study from Iridaceae, Doryanthaceae, Tecophiliaceae, and Xeronemataceae.

Sr.No.	Taxa	Collection sites/regions- Nurseries/Botanical Garden/Institute
1.	*Isophysis tasmanica* T.moore	Plant of Tasmania Nursery, Australia
2.	*Patersonia fragilis* Labill. Asch. & Graebn.	Tasmania, Australia
3.	*Aristea ecklonii* Baker	Burncoose Nursery Gwennap, United Kingdom
4.	*Nivenia stokoei* (Guthrie) N.E.Br.	Western Cape, South Africa
5.	*Witsenia maura* Thunb.	Cape Town, South Africa
6.	*Chasmanthe floribunda* (Salisb.) N.E.Br.	Cistus Nursery, Oregon, USA
7.	*Dierama igneum* Klatt	Digging Dog Nursery, Albion, California
8.	*Tritonia disticha* Klatt (Baker)	Far Reaches Farm, Washington, United States
9.	*Hesperantha coccinea* (Backh & Harv.) Goldblatt & J.C. Manning	Digging Dog Nursery, Albion, California
10.	*Freesia laxa* (Thunb.) Goldblatt & J.C. Manning	Far Reaches Farm, Washington, United States
11.	*Gladiolus communis* L.	SIGNA, Species Iris Group of North America
12.	*Watsonia pillansii* L.Bolus	Hot Plant Company West Sussex, United Kingdom
13.	*Iris koreana* Nakai	Ojeon-ri, Murya-myeon, Bonghwa-gun, Gyeongsangbuk-do, Republic of Korea
14.	*Moraea spathulata* (L.f.) Klatt	Far Reaches Farm, Washington, United States
15.	*Moraea polystachya* (Thunb.) Ker Gawl.	SIGNA, Species Iris Group of North America
16.	*Diplarrena moraea* Labill.	Plant of Tasmania Nursery, Australia
17.	*Sisyrinchium angustifolium* Mill.	Jeju Island, Republic of Korea
18.	*Sisyrinchium idahoense* E.P. Bicknell	Far Reaches Farm, Washington, United States
19.	*Libertia pulchella* R.Br. Spreng.	Plant of Tasmania Nursery, Australia
20.	*Olsynium douglasii* (A.Dietr.) E.P.Bicknell	Far Reaches Farm, Washington, United States
21.	*Trimezia steyermarkii* R.C.Foster	SIGNA, Species Iris Group of North America
22.	*Neomarica candida* (Hassl.) Sprague	SIGNA, Species Iris Group of North America
23.	*Tigridia pavonia* (L.f.) DC.	Far Reaches Farm, Washington, United States
24.	*Cypella coelestis* (Lehm.) Ravenna	SIGNA, Species Iris Group of North America
25.	*Doryanthes palmeri* W.Hill Ex Benth.	Eastern Australia
26.	*Tecophileae violiflora* Bertero ex Colla	LA Serena, Chile, United States
27.	*Xeronema callistemon* W.B.R.Oliv.	Royal Botanic Gardens, Kew, Richmond, UK

### 2.2 Genome sequencing, assembly, and annotation

Libraries were sequenced using the Illumina MiSeq sequencing platform (Illumina, Seoul, Korea). Each sample produced at least 1 GB of raw data. The average length of the paired-end reads (raw reads) was 301 bp. Before assembly, the raw reads were trimmed to sequences with more than 3% probability of error per base using Geneious prime 2020.1.2 (Kearse et al., 2012) to exclude poor-quality reads. The cp genome of *Iris sanguinea* (GenBank accession KT626943) was used as a reference for assembled reads by performing a “map to reference”. The isolated reads were then extracted and reassembled using the option “*De novo* Assembly” in Geneious prime 2020.1.2 (Kearse et al., 2012), followed by the completion of this step until quadripartite structures were obtained. We checked and analyzed the gene boundaries for each protein-coding gene by using the *Iris sanguinea* plastome as a reference with default settings using 80% similarity to annotate genes. The assembled plastid genomes were annotated using GeSeq (Tillich et al., 2017), and all tRNAs were set using tRNAScan-SE (Chan and Lowe, 2019) by default search. Full circular maps were generated using OGDRAW software (Greiner et al., 2019).

### 2.3 Comparative genomic analysis

The 24 complete plastid genome sequences ranging from 150,062 to 164,622 bp were compared and aligned in MAFFT (Kearse et al., 2012) and visualised in an mVISTA plot using Shuffle-LGAN mode (Frazer et al., 2004). *Iris loczyi* was used as the reference genome. The nucleotide diversity (Pi values) of protein-coding genes and RNA genes (tRNA and rRNA) among Iridaceae species was calculated using the DnaSP 6.0 program (Rozas et al., 2017). Any major structural changes, including junctions, boundaries (LSC/IRs and SSC/IRs), and IR expansions and contractions, were investigated using IRscope (Amiryousefi et al., 2018).

### 2.4 Relative synonymous codon usage analysis and selective pressure estimation

The relative synonymous codon usage (RSCU) was examined from the 79 protein-coding genes using DAMBE program to determine the comparative inclination of a specific base encoding the necessary amino acid codon (Xia and Xie, 2001). The values of RSCU greater than one were regarded as having a higher codon frequency.

The synonymous (Ka) and non-synonymous (Ks) substitution ratios of *ycf*2 gene were calculated using the KaKs calculator (http://services.cbu.uib.no/tools/kaks). The average K_a_/K_s_ of *ycf*2, however, was >1, indicating positive selection. Thereafter, selective pressure estimation was carried out to investigate positive selection on the *ycf*2 gene of 12 species covering three subfamilies. The *ycf*2 sequences were individually extracted and aligned using MEGA6 (Tamura et al., 2013). Positive selection was identified using the branch-site model in the codeml program, which was implemented in the PAML v4.9 package (Yang, 2007). The codon frequencies were predicted using the F3 × 4 model. Selective pressure is calculated by the ratio (ω) of the nonsynonymous substitution rate (dN) to the synonymous substitutions rate (dS) which is used in most adaptive evolutionary studies. In this study, there were three sets of comparison models: M0 (single ratio) and M3 (discrete), M7 (beta) and M8 (beta and ω), and M1 (near neutral) and M2 (selection), which were used to find the sites under positive selection (ω > 1) in the *ycf*2 gene. An amino acid site with posterior probabilities >0.95 was considered as positively selected.

### 2.5 Phylogenomic analysis

A phylogenetic analysis of 42 plastid genome sequences was performed. In this study, 31 ingroup taxa from Iridaceae and three outgroup taxa (Doryanthaceae, Tecophiliaceae, and Xeronemataceae) were newly sequenced, and the data for the remaining species were downloaded from NCBI GenBank. To strengthen the representation of the closely related Asparagales family, 11 outgroup taxa were included based on previous phylogenetic studies (Chen et al., 2013). We extracted 79 protein-coding genes and used MAFFT version 7.309 to align each gene individually. Then, we analyzed the alignment and adjusted it manually using Geneious Prime (Kearse et al., 2012). The combined alignment matrix comprised 42 taxa and 71,829 characters.

Maximum parsimony (MP), maximum likelihood (ML), and Bayesian inference (BI) analyses were performed for phylogenetic analysis. MP analysis was performed using PAUP* version 4.0 (Wilgenbusch and Swofford, 2003) using comparable character allowance, and omitted data were treated as gaps. Tree bisection and reconnection branch swapping were used for searches of 1,000 randomly chosen additions, and MulTree allowed 10 trees to be held at each stage. Bootstrap analyses (1,000 pseudoreplicates) were performed to determine the relative bootstrap support with similar parameters. We utilized jModel test version 2.1.7 for ML and BI (Darriba et al., 2012) analyses to obtain suitable models based on the Akaike information criterion and selected GTR+I+G as the best model. ML analysis was conducted using IQ-TREE (Trifinopoulos et al., 2016), and support values were calculated with 1,000 bootstrap replicates. BI analysis was performed using MrBayes (Ronquist et al., 2012). Trees were sampled every 1,000 generations during two independent runs of random trees with a minimum of 1 × 10^6^ generations. The first 25% of the trees were removed as burn-in, and the remaining trees were used to build a consensus tree with 50% majority-rule, with the proportion of bifurcations in the consensus tree provided as a posterior probability (PP) to evaluate the robustness of half of the BI tree. The model parameters were then verified using the effective sample size values. Finally, phylogenetic trees were modified using Figtree program (Rambaut, 2020).

## 3 Results

### 3.1 Plastid genome organization and gene features

**Figure 1:**
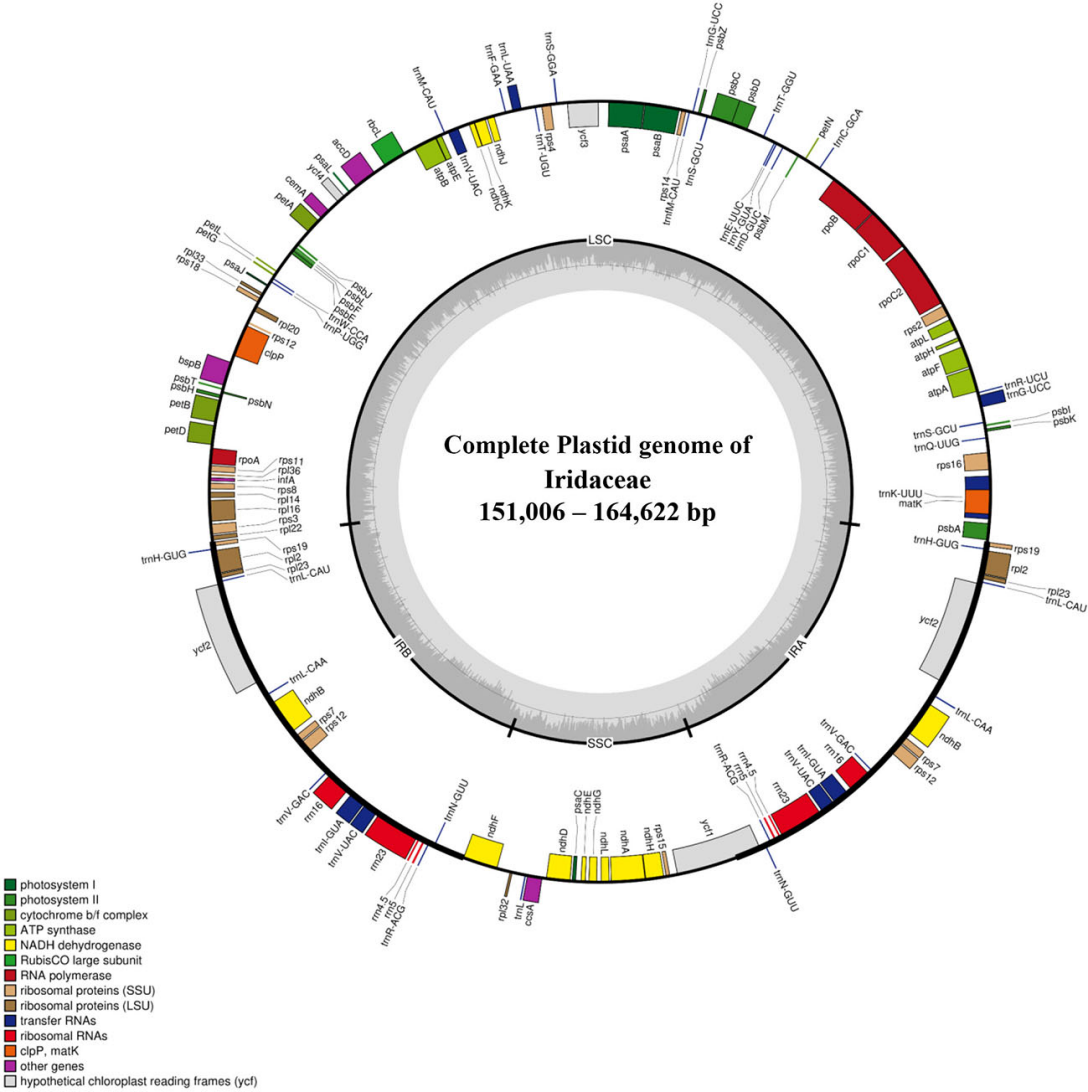
Representative chloroplast genome of Iridaceae. The colored bars indicate protein-coding genes, tRNA genes, and rRNA genes. Genes drawn outside of the map circle are transcribed clockwise, while that drawn inside are transcribed counterclockwise. The darker gray in the inner circle corresponds to the GC content, while the lighter gray corresponds to the AT content.

The plastid genomes of 24 species of Iridaceae, including representative taxa from the subfamilies Isophysioideae, Patersonioideae, Aristeoideae, Nivenioideae, Crocoideae, and Iridoideae, were constructed. The genomes of additional species, including *Crocus* spp., *Iris* spp., and subfamily Geosirioideae (*Geosiris australiensis* and *G. aphylla*), were obtained from NCBI GenBank. All the newly assembled contigs formed a quadripartite structure. The resultant plastomes were composed of aLSC, SSC, and a pair of inverted repeats (IRa and IRb) (**Figure 1**). The size of the CP genomes varied from 119,004 to 164,622 bp (**Table 1**). Among the seven Iridaceae subfamilies, Geosirioideae had the smallest cpDNA (*Geosiris australiensis* NC_042825; 119,004 bp) and the largest cpDNA (*Tigridia pavonia*; 164,622 bp). In addition, the GC content of the cpDNA sequence in Iridaceae was approximately 38% for all the examined taxa. The plastid genome of the autotrophic Iridaceae comprised 113 genes, including 79 protein-coding genes, 30 tRNA genes, and four rRNA genes distributed in the IR region (**Table 2**). Among them, 18 were intron-containing genes, including six tRNA and 12 protein-coding genes. Three of the latter genes—clpP, *rps*12, and *ycf*3—contained two introns. The 5′ exon of the trans-spliced *rps*12 gene is located in the LSC area, whereas the 3′ exon and intron are located in the IR regions. In addition, significant inversions and variable INDEL patterns of resident pseudogenes were found in some Iridaceae species.

**Table 2:** Comparison of the features of plastomes from representative taxa of Iridaceae.

Subfamily	Taxa	Length (bp) and GC content (%)				Total No. of genes/pseudogene	GenBankAccession no.
		Total	LSC	SSC	IR		
**Isophysidoideae**	*Isophysis tasmanica*	158,654 (37.7)	87,184 (35.9)	18,200 (31.6)	26,635 (42.8)	79/0	OP729163
**Patersonioideae**	*Patersonia fragilis*	155,062(37.8)	84,038 (35.9)	17,978 (31.2)	26,523 (42.9)	79/0	OP729169
**Aristeoideae**	*Aristea ecklonii*	153,548(37.6)	83,036 (35.7)	17,414 (31.1)	26,549 (42.6)	79/0	OP710243
**Nivenioideae**	*Nivenia stokoei*	152,780(37.8)	82,549 (35.9)	17,989 (31.3)	26,121 (43.1)	79/1	OP729167
	*Witsenia maura*	151,260(37.8)	81,377 (35.9)	17,533 (31.4)	26,175 (43.0)	79/0	OP745524
**Geosiridaceae**	*Geosiris aphylla*	123,620(38.0)	59,181 (35.1)	12,428 (30.4)	25,293 (43.3)	62/38	MH251635
	*Geosiris australiensis*	119,004(38.5)	45,795(35.8)	515 (31.7)	36,347 (40.3)	50/23	NC_042825
**Crocoideae**	*Chasmanthe floribunda*	151,293 (37.7)	81,003 (35.8)	17,410 (31.1)	26,440 (42.8)	79/1	OP710244
	*Dierama igneum*	152,328 (37.7)	81,657 (35.9)	17,695 (31.1)	26,488 (42.7)	79/0	OP710246
	*Tritonia disticha*	151,724 (37.7)	80,993 (35.9)	17,725 (30.9)	26,503 (42.7)	79/0	OP745522
	*Hesperantha coccinea*	151,959 (37.5)	81,488 (35.6)	17,945 (30.6)	26,263 (42.9)	79/1	OP729160
	*Crocus cartwrightianus*	150,911 (37.5)	81,405 (35.5)	17,392 (30.8)	26,057 (42.8)	79/0	MH542232
	*Crocus sativus*	150,820 (37.5	81,310 (35.6)	17,396 (30.8)	26,057 (42.8)	79/0	NC_041460
	*Freesia laxa*	152,858 (37.6)	82,065 (35.7)	17,817 (31.0)	26,488 (42.7)	79/0	OP710248
	*Gladiolus communis*	151,608 (37.7)	81,516 (35.8)	17,868 (31.0)	26,112 (43.0)	79/0	OP729159
	*Watsonia pillansii*	151,981 (37.7)	81,959 (35.8)	18,120 (31.1)	25,951 (42.9)	79/0	OP745523
**Iridoideae**	*Iris domestica*	153,735 (37.9)	83,199 (36.0)	18,168 (31.5)	26,184 (43.1)	79/0	MW039136
	*Iris gatesii*	153,441 (37.9)	82,702 (36.0)	18,371 (31.5)	26,184 (43.1)	79/0	KM014691
	*Iris koreana*	150,852 (37.9)	81,566 (36.1)	18,286 (31.3)	25,500 (43.1)	79/0	OP729162
	*Iris sanguinea*	152,408 (38.0)	82,340 (36.2)	18,016 (31.8)	26,026 (43.1)	79/0	KT626943
	*Moraea spathulata*	152,127 (37.8)	81,440 (36.0)	18,467 (31.2)	26,110 (43.1)	79/0	OP729166
	*Moraea polystachya*	152,361 (37.9)	81,862 (36.0)	18,201 (31.3)	26,149 (43.1)	79/0	OP729165
	*Diplarrena moraea*	155,895 (37.7)	84,871 (35.8)	18,256 (31.0)	26,384 (43.0)	79/1	OP710247
	*Sisyrinchium angustifolium*	151,305 (37.6)	80,790 (35.6)	18,139 (30.5)	26,188 (43.1)	79/0	OP745518
	*Sisyrinchium idahoense*	151,006 (37.6)	80,417 (35.6)	18,269 (30.5)	26,160 (43.1)	79/0	OP745519
	*Libertia pulchella*	154,129 (37.6)	82,922 (35.7)	18,261 (30.9)	26,473 (43.0)	79/0	OP729164
	*Olsynium douglasii*	152,197 (37.3)	82,087 35.3	18,168 (30.4)	25,971 (42.9)	79/0	OP729168
	*Trimezia steyermarkii*	152,782 (37.7)	82,290 (35.9)	18,154 (31.3)	26,169 (42.9)	79/1	OP745521
	*Neomarica candida*	151,102 (37.6)	82,578 (35.7)	18,424 (31.0)	25,050 (43.1)	79/0	OP729161
	*Tigridea pavonia*	164,622 (38.4)	82,723 (36.0)	18,575 (31.3)	31,662 (43.5)	79/0	OP745520
	*Cypella coelestis*	152,789 (37.8)	82,123 (36.0)	18,526 (31.2)	26,070 (43.0)	79/0	OP710245

### 3.2 Pseudogenization and inversions

**Figure 2:**
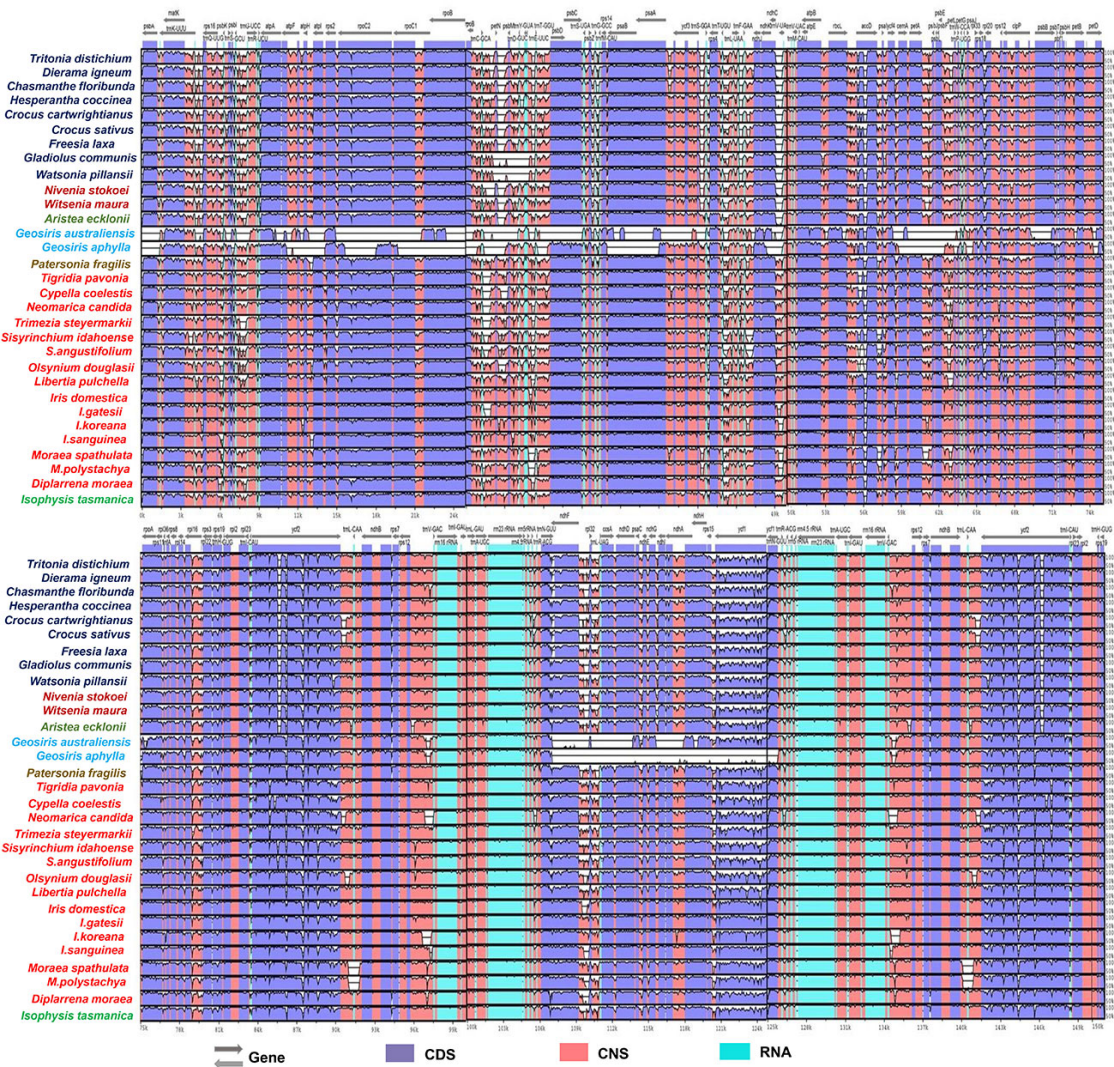
Sequence identity plot comparing 31 CP genomes with *I. loczyi* as a reference using mVISTA. CDS, coding sequences; CNS, non-coding sequences.

Although the overall pattern of genes was similar between species, we found that several protein-coding genes with frameshifts, substitutions and point mutations, internal stop codons, and unclear start codons were classified as pseudogenes. Several genes from the examined species had pseudogenization annotated as *ndh*G (*Diplarrena moraea* and *Nivenia stokoei*), *ndh*D (*Hesperantha coccinea*), *rpo*C2 (*Gladiolus communis*), rpoB (*Chasmanthe floribunda*), *ndh*K (*Witsenia maura*), and *ycf*1 (*Trimezia steyermarkii*) (**Table 2**). Based on the mVISTA data, a detailed comparison study was performed, as demonstrated in **Figure 2**, where there were similarities in coding genes; however, some differences among Iridaceae species were identified. We observed several structural variations, such as inversions in genome structure among species. Unlike other Iridaceae species, *Moraea polystachya* and *M. spathulata* possessed intergenic spacer inversions from *ycf*2 to *ndh*B (approximately 1.7 kb). In addition, *Watsonia longifolia* and *Gladiolus communis* exhibited significant inversions from *pet*N to *trn*E-UUC (approximately 3 kb) (**Figure 2**).

In many studies, *ycf*2 gene alone provides well-supported phylogeny because of its large size and extensive sequence variation (containing many INDELS) uniquely shared by some species and is frequently employed in phylogenetic research on angiosperms (Huang et al., 2010; Zhong et al., 2019; Tang et al., 2022). Our findings revealed significant deletions and inconsistent variations in the *ycf2* gene region. The *ycf2* gene of Iridaceae species was compared, and it was observed that the subfamilies Crocoideae, Nivenioideae, and Aristeoideae had 237 base sequence deletions in the same locus (**Figure 2**; **Supplementary Figure S1**). Similar deletion events may provide a plausible explanation for the sister relationships among these three subfamilies. To examine the existence of *ycf*2 gene based on selective pressure (positive or purifying selection), we generated the K_a_/K_s_ (synonymous and non-synonymous) ratio values of the representative taxon of Iridaceae (**Supplementary Table S4**—**4.1**). Thereafter, the average K_a_/K_s_ of *ycf2*, however, was >1, indicating positive selection.

The PAML v4.9 package (Yang, 2007) was used to measure the logarithmic likelihood value (InL) and estimation of positive selection for the complete sequence data of the *ycf*2 protein-coding region of 12 species in the Iridaceae family. There were several positive selection loci found in models M2 (positive selection) and M8 (beta and w>1) with the posterior probability superior to 95% and 99% as calculated by Bayes empirical and naïve empirical estimation (**Supplementary Table S4**—**4.2**).

### 3.3 Nucleotide diversity and IR boundaries

**Figure 3:**
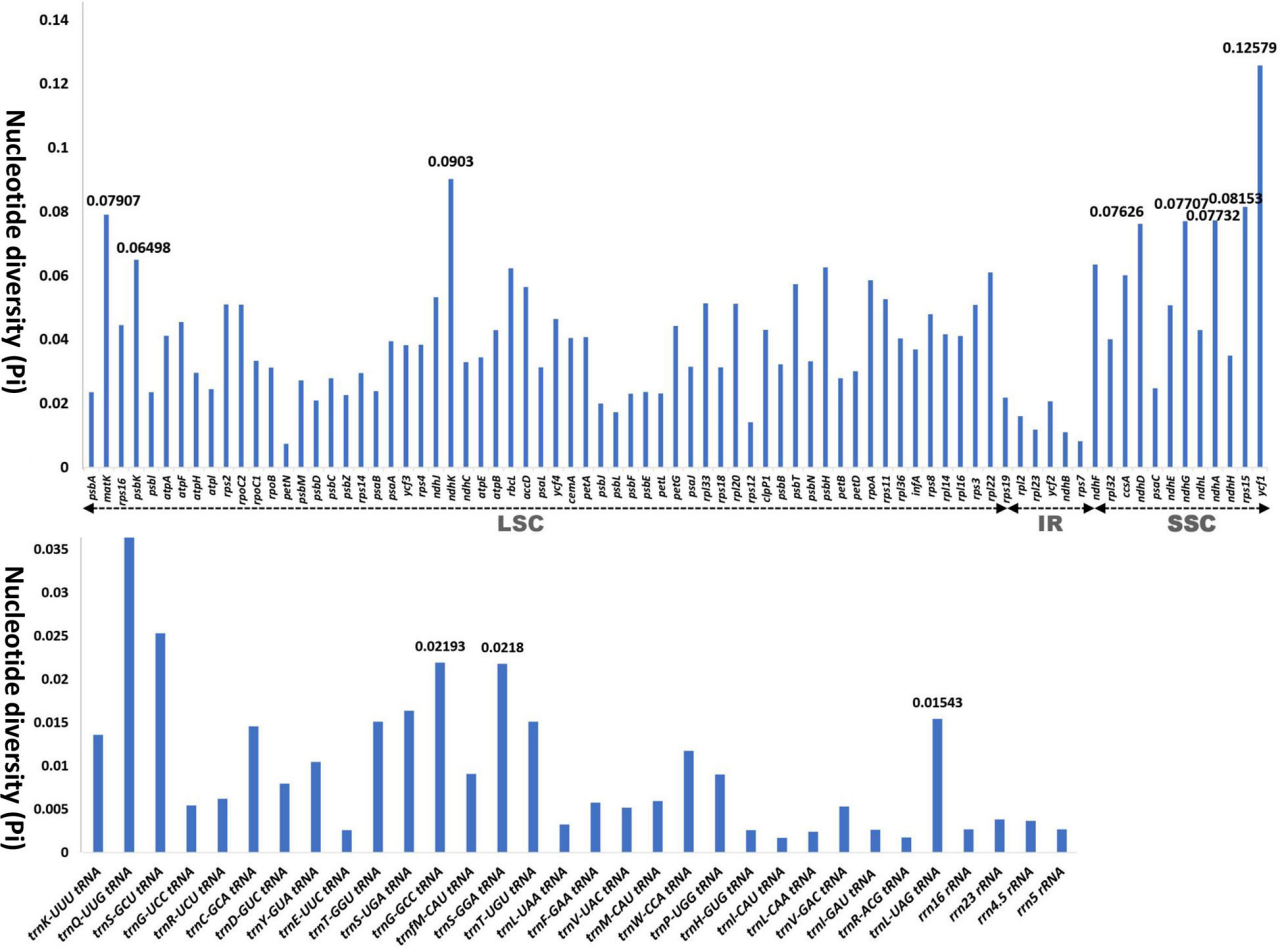
Graphs showing the variation in nucleotide diversity (Pi) in the protein-coding region, transfer RNA, and ribosomal RNA in 24 representative taxa of Iridaceae.

We analyzed the nucleotide diversity of protein-coding genes, tRNA, and rRNA of the 31 ingroup taxa comprising seven subfamilies of Iridaceae to identify phylogenetically informative genes (**Figure 3**; **Supplementary Table S1**). In total, 113 genes from different regions, that is, IR, LSC, and SSC, were aligned to calculate pi values. The nucleotide diversity of the IR region in protein-coding genes was relatively low compared with that of the LSC and SSC regions. In the CDS, the highest pi values were recorded for *mat*K (0.07907), followed by NADH dehydrogenase genes (*ndh*K, *ndh*F, *ndh*D, and *ndh*G), *ycf*1, and *rps*15. Among RNAs, the pi value ranged from 0.04019 (*trn*Q-UUG) to 0.00267 (*rrn*16rRNA and *rrn*5rRNA), whereas two genes, *trn*A-UGC and *trn*N-GUU, exhibited no nucleotide diversity (Pi value: 0) (**Figure 3**). The detailed values are shown in **Supplementary Table S1**.

**Figure 4:**
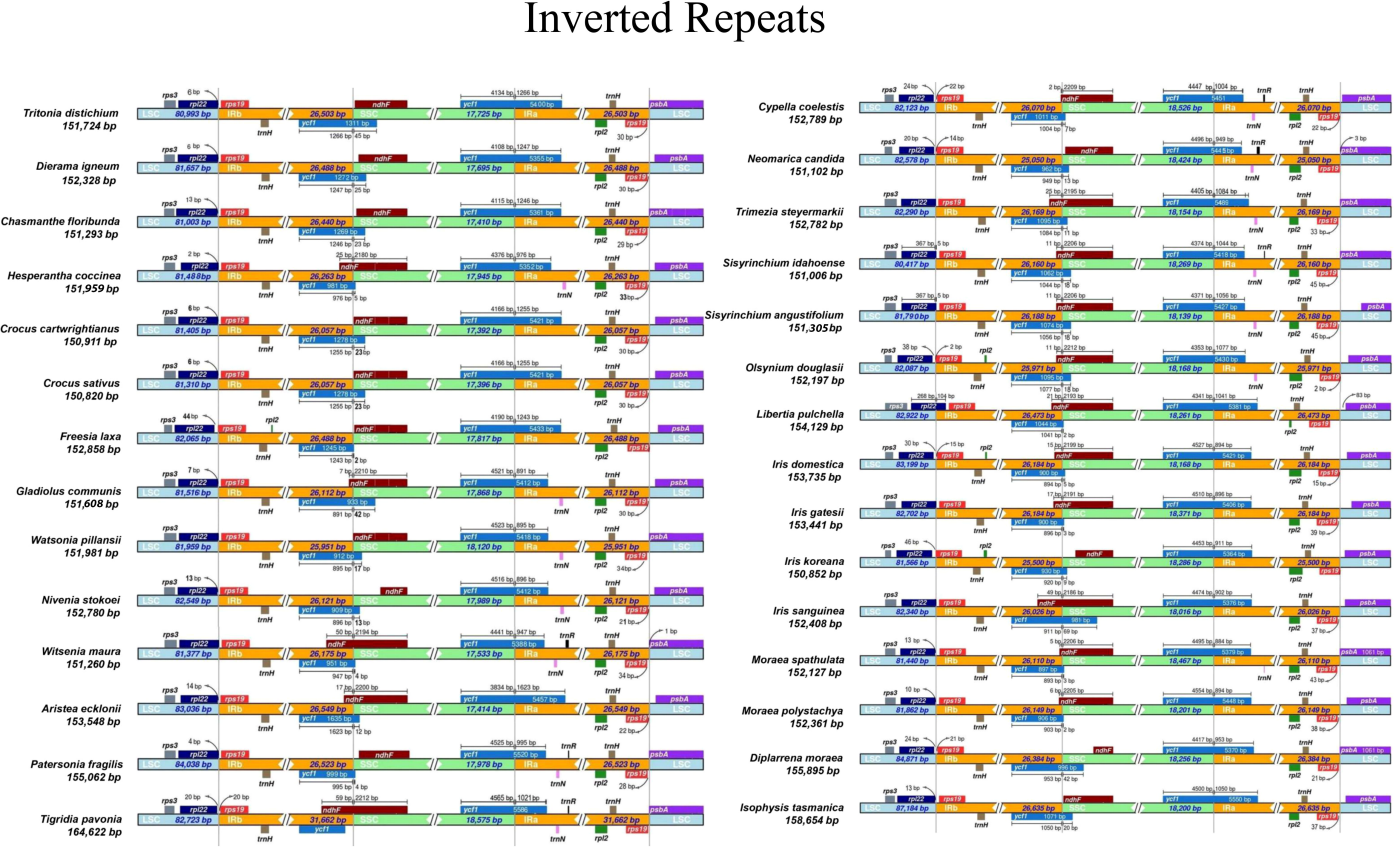
Comparison of the borders of large single copy, small single copy, and inverted repeat regions of chloroplast genomes of Iridaceae.

The primary cause of size differences in cp genomes is the contraction and extension of IR boundaries, which are frequent evolutionary events (Wang et al., 2008; Zong et al., 2019). The boundaries (LSC/IRs and SSC/IRs) were similar among Iridaceae species (**Figure 4**). Despite the boundary of plastome sequences of the Iridaceae species being largely constant, we found expanded IR regions, whereas the *rpl22* gene in the LSC region was lengthened by 104 bp only in *Libertia pulchella*. A partially duplicated copy of the *ycf*1 gene was observed at the IRb–SSC junctions of all plastid genomes. Furthermore, *rps*19 gene was duplicated in the IR region. Of these, *Tigridia pavonia* presented a large genome, owing to the enlarged IR length. Notably, the Australian species *Patersonia fragilis*, *Isophysis tasmanica*, and *Diplarrena moraea* had LSC with more base pairs than the other species (**Figure 4**) due to INDEL mutations of nucleotides in the non-coding region.

### 3.4 Relative synonymous codon usage assembly

**Figure 5:**
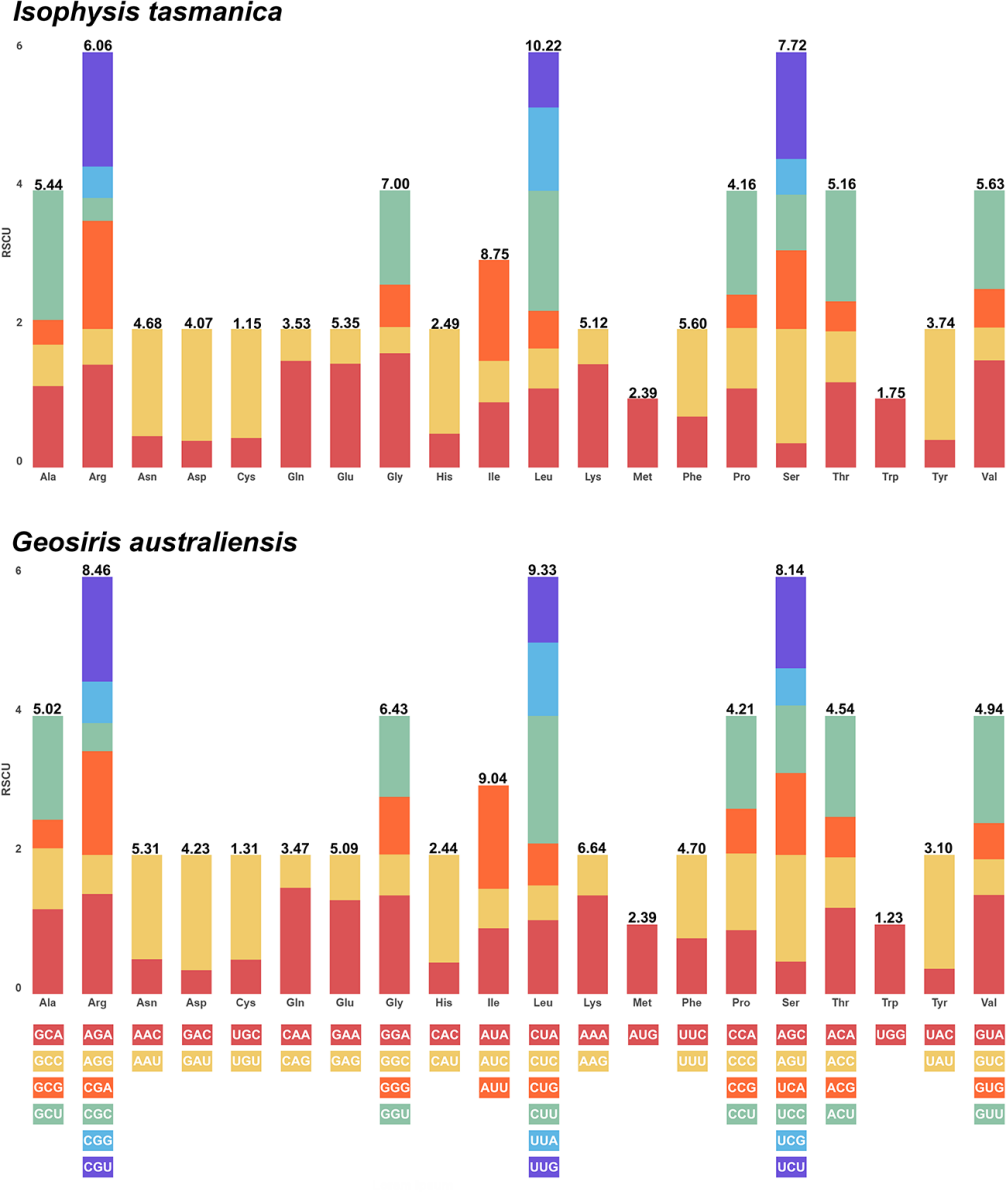
Number of codons and relative synonymous codon usage values of the plastid genome of representative autotrophic (*Isophysis tasmanica*) and mycoheterotrophic (*Geosiris australiensis*) species of Iridaceae.

A total of 79 protein-coding genes of 31 representative taxa of Iridaceae were used to compare the values of RSCU (**Supplementary Figure S4**). The stop codons UAA, UAG, and UGA were excluded. The total observed frequency ranged from 22,805 (*Isophysis tasmanica*) to 6,194 (*Geosiris australiensis*) (**Figure 5**). Leucine (L) was the most prevalent amino acid in the autotrophic Iridaceae (10.25 to 11.08%), whereas isoleucine (I) and leucine (L) were the most extensive amino acid in the mycoheterotrophic species (*Geosiris*). Notably, cysteine (C) was the least abundant amino acid in both photosynthetic and non-photosynthetic groups (1.09 to 1.20%) (**Supplementary Figure S4**, **Supplementary Table S5**). Both methionine (M) and tryptophan (W) show an RSCU value (equal to 1) that exhibited no codon preferences in all the examined species.

### 3.5 Phylogenetic analysis

**Figure 6:**
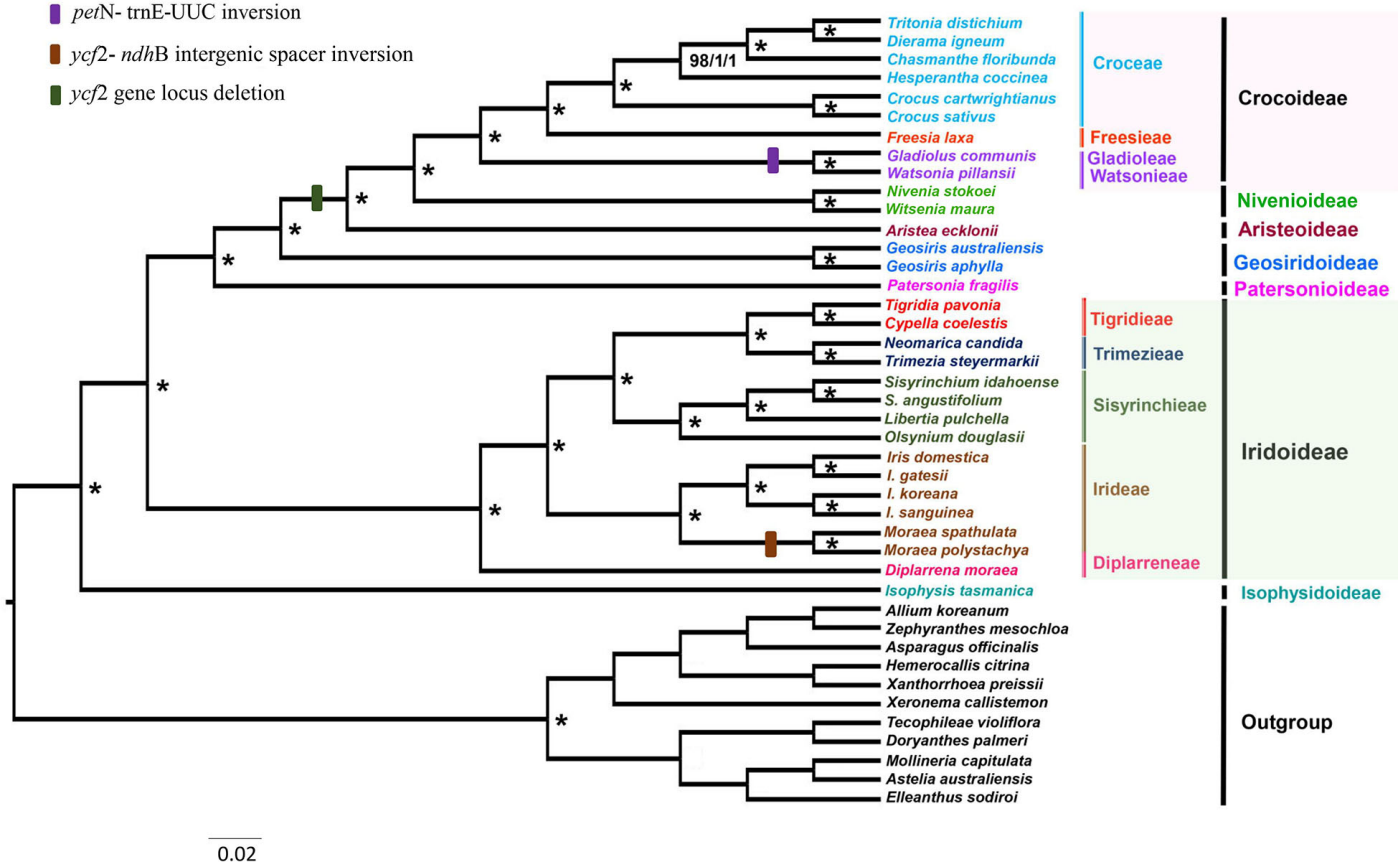
Estimated phylogenetic tree of Iridaceae based on maximum parsimony (MP), maximum likelihood (ML), and Bayesian inference (BI) analyses using 79 protein-coding genes. The asterisks (*) around nodes indicate a bootstrap value of 100% for MP and ML analyses and a posterior probability of 1.0 for BI analysis.

The phylogenetic reconstructions based on 79 protein-coding genes for 31 ingroup (Iridaceae) and 11 outgroup species contained 71,829 characters, resulting in phylogenies with identical topologies using MP, ML, and BI with bootstrap values of 100% and PP of 1.0 (**Figure 6**). Iridaceae was recovered as monophyletic with strong support (100%, 1) as in previous studies performed by Goldblatt on the basis of the selected genes.

This study, with relatively more coding gene sequences than previous molecular studies, represents the extent of relationships within Iridaceae. The evolutionary relationships found in this study at the subfamily level were notably consistent with prior extensive investigations (Isophysioideae, *Isophysis tasmanica*, sister to other Iridaceae species, 100%, 1). The three subfamilies Patersonioideae, Geosirioideae, and Aristeoideae comprised a well-supported clade (100%, 1), together with the two subfamilies Crocoideae and Nivenioideae (100%, 1). *Geosiris australiensis* and *G. aphylla* exhibited substantial plastome rearrangements and gene losses relative to other Iridaceae species, but they were nonetheless highly supported as a clade (100%, 1). *Diplarrena moraea* was sister to other genera in the Iridoideae clade. Diplarreneae was further subdivided into two distinct clades: one Irideae and three important tribal clusters, including Sisyrinchieae, sister to Trimezieae, and the remaining Tigrideae with high support values in MP, ML, and BI analyses (**Figure 6**).

## 4 Discussion

### 4.1 Plastome structure and comparisons

Our first comprehensive representative plastome sampling from the basal clade Isophysioideae to the higher Crocoideae clade offers an excellent prospect for investigating plastome evolution. Overall, the plastome lengths in the photosynthetic group ranged between 150,820 and 164,622 bp (**Table 1**). The LSC and SSC regions are comparatively longer with a higher AT content than the IR region, with the notable exception of the plastomes in the non-photosynthetic group, which contain significantly reduced SSC and extended IR regions (*e*.*g*., *Geosirioideae*) wherein photosynthetic genes have been extensively dropped (Joyce et al., 2018). In the evolution of land plants for both autotrophic and heterotrophic taxa, substantial extension of the IR region has occurred several times through SSC gene transporters (Naumann et al., 2016; Zhu et al., 2016; Sinn et al., 2018). Notably, the published chloroplast genome of *Sisyrinchium angustifolium* (Kang et al., 2020) contained gaps or missing nucleotide bases (129) between the sequences; however, our newly sequenced genome of this species substantially filled the existing gaps using NGS data.

### 4.2 Pseudogenization and sequence inversion

Larger genes encoding protein products (up to 1,000 amino acids) have been demonstrated to be more likely to undergo pseudogenization (Khachane and Harrison, 2009). Events such as INDEL and pseudogenization are also thought to be critical for understanding the evolutionary history (van der Burgt et al., 2014). Several protein-coding genes from the examined Iridaceae species exhibited pseudogenization (**Table 3**)—for instance, in *Trimezia steyermarkii*, the *ycf*1 gene contained several internal stop codons due to INDELS. In *Gladiolus communis*, *rpo*C2 only had one mutation [GAA (glutamate) → TAA (stop codon)], which was recognized as a pseudogene. We likewise identified *ndh*K as a pseudogene in *Witsenia maura* due to a substitution mutation. We also found that *Hesperantha coccinea* had a dissimilar frontal region of *ndh*D gene (up to 66 bp). The *ndh*G gene was functional in Iridaceae species; however, it underwent pseudogenization by base insertion, changing the reading frame in *Diplarrena moraea.* Within Iridaceae, *Geosiris* species also showed pseudogenization of NADH dehydrogenase and other photosynthetic genes (Joyce et al., 2018). In addition, heterotrophic plants frequently experience *ndh* gene loss and pseudogenization (Wicke and Naumann, 2018), which are also prevalent in other autotrophic plants, including conifers (Wakasugi et al., 1994; Ni et al., 2017), Orchidaceae (Jiang et al., 2022), and Papavaraceae (Xu and Wang, 2021), suggesting that this is a random phenomenon in the evolution of photosynthetic lineages.

**Table 3:** Gene composition within chloroplast genomes of Iridaceae species.

Groups of genes		Names of genes	No.
RNA genes	Ribosomal RNAs	*rrn*4.5 ^x2^, *rrn*5 ^x2^, *rrn*16 ^x2^, *rrn*23 ^x2^	8
	Transfer RNAs	*trn*K-UUU^a^, *trn*Q-UUG, *trn*S-GCU, *trn*G-UCC^a^, *trn*R-UCU, *trn*C-GCA, *trn*D-GUC, *trn*Y-GUA, *trn*E-UUC, *trn*T-GGU, *trn*S-UGA, *trn*G-GCC, *trnf*M-CAU, *trn*S-GGA, *trn*T-UGU, *trn*L-UAA^a^, *trn*F-GAA, *trn*V-UAC^a^, *trn*M-CAU, *trn*L-UAG, *trn*W-CCA, *trn*P-UGG, *trn*H-GUG ^x2^, *trn*I-CAU ^x2^, *trn*L-CAA ^x2^, *trn*V-GAC ^x2^, *trn*I-GAU^a x2,^ *trn*A-UGC^a x2^, *trn*R-ACG ^x2^, trnN-GUU ^x2^	38
Protein genes	Photosystem I	*psa*A, *psa*B, *psa*C, *psa*I, *psa*J	5
	Photosystem II	*psb*A, *psb*B, *psb*C, *psb*D, *psb*E, *psb*F, *psb*H, *psb*I, *psb*J, *psb*K, *psb*L, *psb*M, *psb*N, *psb*T, *psb*Z	15
	Cytochrome	*pet*A, *pet*B^a^, *pet*D^a^, *pet*G, *pet*L, *pet*N	6
	ATP synthases	*atp*A, *atp*B, *atp*E, *atp*F^a^, *atp*H, *atp*I	6
	Large unit of Rubisco	*rbc*L	1
	NADH dehydrogenase	*ndh*A^a^, *ndh*B^ax2^, *ndh*C, *ndh*D^(Ψ)^, *ndh*E, *ndh*F, *ndh*G^(Ψ)^, *ndh*H, *ndh*I, *ndh*J, *ndh*K^(Ψ)^	12
	ATP-dependent protease subunit P	*clp*P^b^	1
	Envelope membrane protein	*cem*A	1
Ribosomal proteins	Large units of ribosome	*rpl*2^a x2^, *rpl*14, *rpl*16^a^, *rpl*20, *rpl*22, *rpl*23 ^x2^, *rpl*32, *rpl*33, *rpl*36	11
	Small units of ribosome	*rps*2, *rps*3, *rps*4, *rps*7 ^x2^, *rps*8, *rps*11, *rps*12^b x2^, *rps*14, *rps*15, *rps*16^a^, *rps*18, *rps*19 ^x2^	15
Transcription/ translation	RNA polymerase	*rpo*A, *rpo*B^(Ψ)^, *rpo*C1^a^, *rpo*C2^(Ψ)^	4
	Initiation factor	*inf*A	1
	Miscellaneous protein	*acc*D, *ccs*A, *mat*K	3
	Hypothetical proteins and conserved reading frames	*ycf*1^(Ψ)^, *ycf*2 ^x2^, *ycf*3^b^, *ycf*4	5
Total			132

^a^: gene with one intron; ^b^: gene with two introns; ^x2^: duplicated gene; ^Ψ^: pseudogene in *Diplarrena moraea* and *Nivenia stokoei*, *Gladiolus communis*, *Hesperantha coccinea*, *Chasmanthe floribunda*, *Trimezia steyermarkii*, *Witsenia maura*.

Studies have shown that plastomes in many angiosperm lineages have genomic structural differences, called inversions, ranging from small to large inversions, which provide important phylogenetic information (**Figure 2**). These include Onagraceae (Hupfer et al., 2000), Asteraceae (Walker et al., 2014), Fabaceae (Wang et al., 2018; Charboneau et al., 2021), and Commelineaceae (Jung et al., 2021). Previously, Jung et al. (2021) reported that AT-rich sequences caused genomic rearrangements, such as inversions. Our results also identified two inversion events, *ycf*2-*ndh*B intergenic region inversion, in two *Moraea* species, *M. spathulata* and *M. polystachya*, that showed high AT-rich content due to weak hydrogen bonding (**Supplementary Figures S2**, **S3**). Unlike other Iridaceae species, *Watsonia* and *Gladiolus* have one large inversion (*pet*N-*trn*E*-*UUC), and both species have reversed orientation in the same loci, demonstrating high AT content and forming a clade sister in Crocoideae. Therefore, genome sequence comparisons are appropriate for analyzing structural variations.

### 4.3 Gene locus deletion in *ycf*2 gene

The *ycf*2 gene is the largest protein-coding gene reported in flowering plants (Drescher et al., 2000). The function of *ycf*2 gene is unknown, but it does not seem to be specifically linked to photosynthesis (Drescher et al., 2000). In our study, *ycf*2 sequences contained many INDELS that were distinctively shared by some examined species. Members of the Crocoideae, Nivenioideae, and Aristeoideae subfamilies shared a 237-bp deletion in the *ycf*2 gene (**Supplementary Figure S1**) as we moved from the basal (Isophysioideae) to the higher clade (Crocoideae, Nivenioideae, and Aristeoideae) (**Figure 6**). Zhong et al. (2019) performed selective locus detection of the *ycf2* gene in eight species of the composite family and reported that the *ycf*2 gene has undergone adaptive evolution. Hu et al. (2015); Fan et al. (2018); Zhong et al. (2019), and Wu et al. (2020) investigated the positive selection of the protein-coding *ycf*2 gene and reported its role in adaptation to diverse ecosystems. In our study, the gene *ycf*2 was subjected to a selective pressure test using a sequence alignment of *ycf*2 gene that codes for protein to estimate the K_a_/K_s_ ratio, and the results showed K_a_/K_s_ >1, indicating positive selection (**Supplementary Table S4**—**4.1**). Furthermore, *ycf*2 was used for selective pressure estimation using branch-site model, and it was found that the *ycf*2 gene was a positively selected gene with several positive sites (**Supplementary Table S4**—**4.2**). Notably, *ycf*2 showed the greatest degree of differentiation, positive selection, and high conservation, which may promote adaptive evolution. Further extensive investigation and detailed discussion on the adaptive evolutionary analysis of the *ycf*2 gene should be conducted in future studies on Iridaceae.

### 4.4 Nucleotide diversity and relative synonymous codon usage analysis

The nucleotide diversity of CDS, tRNA, and rRNA among the Iridaceae plastomes was evaluated to determine genetic divergence. The IR regions had lower nucleotide diversity than the LSC and SSC regions because of their highly conserved composition. We found a phylogenetically informative dataset composed of *mat*K and *ndh* genes (*ndh*K, *ndh*E, *ndh*D, and *ndh*G), *ycf*1, and *rps*15, which had high values (Pi > 0.07). The genes listed above were further used to analyze phylogenetic relationships (**Figure 3**).

These events in protein-coding sequences have helped us to better understand the evolutionary relationships among Iridaceae species and expand our understanding of plastid genome evolution in angiosperms. Our findings offer new evidence for the evolutionary development and history of the core Iridaceae based on specific inversion events and pseudogenization of some genes, which are confined to some species, in contrast to the rest of the studied species.

Codon usage in plastid protein-coding genes plays an important role to infer the evolutionary forces such as mutation–selection and genetic drift within species (Komar, 2016). Most of the amino acids were encoded by two to six synonymous codons, with the exception of methionine (M) and tryptophan (W). For the codons, the RSCU values >1 indicate that the codon are frequently used, and the values <1 imply that the codon is used less frequently than expected (Sharp and Li, 1986). Our results showed that there were 31 codons in autotrophic species and 29 codons in mycoheterotrophic *Geosiris* that have a high value (>1) and ended with A/U. However, most of the codons with low value (<1) ended with G/C (**Supplementary Figure S4**). The most commonly used codon in Iridaceae was Leucine (Leu), except in non-photosynthetic *Geosiris aphylla* (isoleucine, Ile) due to the substantial plastome rearrangements and gene losses relative to other Iridaceae species.

### 4.5 Phylogenetic relationships

In this study, we used plastid protein-coding sequences to analyze representative sampling datasets of Iridaceae. The comparative trial of phylogenomics using plastid genome sequences significantly supported the monophyly of Iridaceae and was in accordance with recently published results (**Figure 6**). The highly supported phylogenetic relations reconstructed by MP, ML, and BI analyses are consistent with previous studies estimated by Goldblatt et al. (2008) and Joyce et al. (2018). The location of *Watsonia* Mill. is the only point of incongruence; Goldblatt et al. (2008) discovered *Watsonia* with other related species in the tribe Watsonieae with weak support (72%). Notably, Joyce et al. (2018) found *Watsonia* and *Schizorhiza* in one clade with strong support (99%, 1), whereas we recovered a clade comprising *Watsonia* and *Gladiolus* with high support values (100%, 1). *Watsonia pillansii* or Beatrice *Watsonia* is a species of cormous perennial geophyte native to southern Africa (South Africa, Lesotho, and Swaziland) in the Iridaceae family, which is closely related to *Gladiolus* (Chen, 2021). Our analysis described *Gladiolus* and *Watsonia* in one clade and presented a similar inversion in comparison with other Crocoideae members (**Figure 6**). Further studies are needed to revise the position of *Watsonia*.

## 5 Conclusion

This study is the first report of a comparative genomic analysis of the second largest family in Asparagales, revealing significant genomic structural characteristics, nucleotide diversity, pseudogenization–inversion events, and variable INDEL patterns using complete plastid genomes of representative taxa of Iridaceae. In addition, relationships among subfamilies and tribes [except *Tritoniopsis* (Tritoniopsideae)] were determined with high support in the current study using 79 protein-coding genes. Plastome data generated new and robust insights into the phylogeny of *Gladiolus*–*Watsonia* clade (Iridaceae, Crocoideae). The three subfamilies share common *ycf*2 gene locus deletion. The complete plastome investigated in this study will facilitate further research on *Iridaceae* and enhance our understanding of plastome evolution.

## Data availability statement

The names of the repository/repositories and accession number(s) can be found at: National Center for Biotechnology Information (NCBI) Bioproject database under accession number OP710243-OP710248; OP729159-OP729169; OP745518-OP745527 (**Supplementary Table S3**).

## Author contributions

KK: experimentation, formal analysis, data curation and interpretation, preparation of figures and tables, and writing of the original draft. JJ: authored or reviewed the drafts of the paper and approved the final draft. J-HK: project administration, conceptualization, supervision, writing—review, and draft approval. All authors contributed to the article and approved the submitted version.

## Supplementary material

Supplementary Figure 1 Gene locus deletion in *ycf*2 gene shared by the sampled species of three subfamilies—Crocoideae, Nivenioideae and Aristeoideae—of Iridaceae. The white gap corresponds to the missing amino acid sequence.

Supplementary Figure 2 Inversion events.

Supplementary Figure 3 Inversion events.

Supplementary Figure 4 Number of codons and relative synonymous codon usage values in all the protein-coding genes of the plastid genome of 31 representative Iridaceae taxa.

Supplementary Table 1 Nucleotide diversity (pi) of Iridaceae species.

Supplementary Table 2 Assembly information of Iridaceae species analysed in this study using NGS.

Supplementary Table 3 Information of the associated Bio project, GenBank Accession, SRA, and Bio-sample numbers of the Iridaceae species analysed in this study.

Supplementary Table 4 4.1 Non-synonymous and synonymous rates for the protein-coding ycf2 gene. 4.2 Positive selective amino acid loci and parameter estimation in ycf2 of twelve species in the Iridaceae family species.

Supplementary Table 5 Number of codons and relative synonymous codon usage values in 31 representative plastid genomes.

## References

[B1] Amiryousefi A. Hyvönen J. Poczai P. (2018). IRscope: an online program to visualize the junction sites of chloroplast genomes. Bioinformatics 34 (17), 3030–3031. doi: 10.1093/bioinformatics/bty220 29659705

[B2] Angiosperm Phylogeny Group Chase M. W. Christenhusz M. J. Fay M. F. Byng J. W. Judd W. S.. (2016). An update of the Angiosperm Phylogeny Group classification for the orders and families of flowering plants: APG IV. Botanical J. Linn. Soc. 181 (1), 1–20. doi: 10.1111/boj.12385

[B3] Birky C. W. Jr. (1995). Uniparental inheritance of mitochondrial and chloroplast genes: mechanisms and evolution. Proc. Natl. Acad. Sci. 92 (25), 11331–11338. doi: 10.1073/pnas.92.25.11331 8524780 PMC40394

[B4] Chan P. P. Lowe T. M. (2019). “tRNAscan-SE: searching for tRNA genes in genomic sequences,” in Gene prediction (Springer), 1–14. 10.1007/978-1-4939-9173-0_1 PMC6768409 31020551

[B5] Charboneau J. L. Cronn R. C. Liston A. Wojciechowski M. F. Sanderson M. J. (2021). Plastome structural evolution and homoplastic inversions in neo-astragalus (Fabaceae). Genome Biol. Evol. 13 (10), evab215. doi: 10.1093/gbe/evab215 34534296 PMC8486006

[B6] Chen Y. (2021). Adapting watsonia pillansii as a new bedding, container, and perennial garden crop plant: Determine vernalization length and establish a cleaning and micropropagation protocol. Available at: https://hdl.handle.net/11299/225175.

[B7] Chen S. Kim D.-K. Chase M. W. Kim J.-H. (2013). Networks in a large-scale phylogenetic analysis: reconstructing evolutionary history of asparagales (Lilianae) based on four plastid genes. PLoS One 8 (3), e59472. doi: 10.1371/journal.pone.0059472 23544071 PMC3605904

[B8] Christenhusz M. J. Byng J. W. (2016). The number of known plants species in the world and its annual increase. Phytotaxa 261 (3), 201–217-201–217. doi: 10.11646/phytotaxa.261.3.1

[B9] Dahlgren R. Bremer K. (1985). Major clades of the angiosperms. Cladistics 1 (4), 349–368. doi: 10.1111/j.1096-0031.1985.tb00433.x 34965682

[B10] Darriba D. Taboada G. L. Doallo R. Posada D. (2012). jModelTest 2: more models, new heuristics and parallel computing. Nat. Methods 9 (8), 772–772. doi: 10.1038/nmeth.2109 PMC4594756 22847109

[B11] Doyle J. Doyle J. (1987). CTAB DNA extraction in plants. Phytochemical Bull. 19, 11–15.

[B12] Drescher A. Ruf S. Calsa T. Jr. Carrer H. Bock R. (2000). The two largest chloroplast genome-encoded open reading frames of higher plants are essential genes. Plant J. 22 (2), 97–104. doi: 10.1046/j.1365-313x.2000.00722.x 10792825

[B13] Dyer T. (1984). The chloroplast genome: its nature and role in development. Topics Photosynthesis 5, 23–69.

[B14] Fan W.-B. Wu Y. Yang J. Shahzad K. Li Z.-H. (2018). Comparative chloroplast genomics of dipsacales species: insights into sequence variation, adaptive evolution, and phylogenetic relationships. Front. Plant Sci. 9, 689. doi: 10.3389/fpls.2018.00689 29875791 PMC5974163

[B15] Frazer K. A. Pachter L. Poliakov A. Rubin E. M. Dubchak I. (2004). VISTA: computational tools for comparative genomics. Nucleic Acids Res. 32 (suppl_2), W273–W279. doi: 10.1093/nar/gkh458 15215394 PMC441596

[B16] Goldblatt P. (1990). Phylogeny and classification of iridaceae. Ann. Missouri Botanical Garden 77 (4), 607–627. doi: 10.2307/2399667

[B17] Goldblatt P. Manning J. Rudall P. (1998). “Iridaceae,” in Flowering Plants·Monocotyledons (Springer), 295–333.

[B18] Goldblatt P. Rodriguez A. Powell M. Davies J. T. Manning J. C. van der Bank M.. (2008). Iridaceae’out of australasia’? phylogeny, biogeography, and divergence time based on plastid DNA sequences. Systematic Bot. 33 (3), 495–508. doi: 10.1600/036364408785679806

[B19] Greiner S. Lehwark P. Bock R. (2019). OrganellarGenomeDRAW (OGDRAW) version 1.3. 1: expanded toolkit for the graphical visualization of organellar genomes. Nucleic Acids Res. 47 (W1), W59–W64. doi: 10.1093/nar/gkz238 30949694 PMC6602502

[B20] Huang J. L. Sun G. L. Zhang D. M. (2010). Molecular evolution and phylogeny of the angiosperm ycf2 gene. J. Systematics Evol. 48 (4), 240–248. doi: 10.1111/j.1759-6831.2010.00080.x

[B21] Hupfer H. Swiatek M. Hornung S. Herrmann R. Maier R. Chiu W.-L.. (2000). Complete nucleotide sequence of the oenothera elata plastid chromosome, representing plastome I of the five distinguishable euoenothera plastomes. Mol. Gen. Genet. MGG 263 (4), 581–585. doi: 10.1007/PL00008686 10852478

[B22] Hu S. Sablok G. Wang B. Qu D. Barbaro E. Viola R.. (2015). Plastome organization and evolution of chloroplast genes in cardamine species adapted to contrasting habitats. BMC Genomics 16 (1), 1–14. doi: 10.1186/s12864-015-1498-0 25887666 PMC4446112

[B23] Jiang H. Tian J. Yang J. Dong X. Zhong Z. Mwachala G.. (2022). Comparative and phylogenetic analyses of six Kenya polystachya (Orchidaceae) species based on the complete chloroplast genome sequences. BMC Plant Biol. 22 (1), 1–21. doi: 10.1186/s12870-022-03529-5 35387599 PMC8985347

[B24] Joyce E. M. Crayn D. M. Lam V. K. Gerelle W. K. Graham S. W. Nauheimer L. (2018). Evolution of geosiris (Iridaceae): historical biogeography and plastid-genome evolution in a genus of non-photosynthetic tropical rainforest herbs disjunct across the Indian ocean. Aust. Systematic Bot. 31 (6), 504–522. doi: 10.1071/SB18028

[B25] Jung J. Kim C. Kim J.-H. (2021). Insights into phylogenetic relationships and genome evolution of subfamily commelinoideae (Commelinaceae mirb.) inferred from complete chloroplast genomes. BMC Genomics 22 (1), 1–12. doi: 10.1186/s12864-021-07541-1 33794772 PMC8017861

[B26] Kang Y. J. Kim S. Lee J. Won H. Nam G.-H. Kwak M. (2020). Identification of plastid genomic regions inferring species identity from *de novo* plastid genome assembly of 14 Korean-native iris species (Iridaceae). PLoS One 15 (10), e0241178. doi: 10.1371/journal.pone.0241178 33104732 PMC7588056

[B27] Kearse M. Moir R. Wilson A. Stones-Havas S. Cheung M. Sturrock S.. (2012). Geneious basic: an integrated and extendable desktop software platform for the organization and analysis of sequence data. Bioinformatics 28 (12), 1647–1649. doi: 10.1093/bioinformatics/bts199 22543367 PMC3371832

[B28] Khachane A. N. Harrison P. M. (2009). Strong association between pseudogenization mechanisms and gene sequence length. Biol. direct 4 (1), 1–5. doi: 10.1186/1745-6150-4-38 19807910 PMC2768697

[B29] Kim J.-H. Kim D.-K. Forest F. Fay M. F. Chase M. W. (2010). Molecular phylogenetics of ruscaceae sensu lato and related families (Asparagales) based on plastid and nuclear DNA sequences. Ann. Bot. 106 (5), 775–790. doi: 10.1093/aob/mcq167 20929900 PMC2958784

[B30] Komar A. A. (2016). The yin and yang of codon usage. Hum. Mol. Genet. 25 (R2), R77–R85. doi: 10.1093/hmg/ddw207 27354349 PMC6372012

[B31] Metelerkamp W. Sealy J. (1983). Some edible and medicinal plants of the doorn karoo. Veld Flora 69 (1), 4. doi: 10.10520/AJA00423203_1247

[B32] Naumann J. Der J. P. Wafula E. K. Jones S. S. Wagner S. T. Honaas L. A.. (2016). Detecting and characterizing the highly divergent plastid genome of the nonphotosynthetic parasitic plant hydnora visseri (Hydnoraceae). Genome Biol. Evol. 8 (2), 345–363. doi: 10.1093/gbe/evv256 26739167 PMC4779604

[B33] Nemati Z. Harpke D. Gemicioglu A. Kerndorff H. Blattner F. R. (2019). Saffron (Crocus sativus) is an autotriploid that evolved in Attica (Greece) from wild crocus cartwrightianus. Mol. Phylogenet. Evol. 136, 14–20. doi: 10.1016/j.ympev.2019.03.022 30946897

[B34] Ni Z. Ye Y. Bai T. Xu M. Xu L.-A. (2017). Complete chloroplast genome of pinus massoniana (Pinaceae): Gene rearrangements, loss of ndh genes, and short inverted repeats contraction, expansion. Molecules 22 (9), 1528. doi: 10.3390/molecules22091528 28891993 PMC6151703

[B35] Rambaut A. (2020). FigTree v. 1.4. 42018 (Google Scholar).

[B36] Reeves G. Chase M. W. Goldblatt P. Rudall P. Fay M. F. Cox A. V.. (2001). Molecular systematics of iridaceae: evidence from four plastid DNA regions. Am. J. Bot. 88 (11), 2074–2087. doi: 10.2307/3558433 21669639

[B37] Ronquist F. Teslenko M. van der Mark P. Ayres D. L. Darling A. Höhna S.. (2012). MrBayes 3.2: efficient Bayesian phylogenetic inference and model choice across a large model space. Systematic Biol. 61 (3), 539–542. doi: 10.1093/sysbio/sys029 PMC3329765 22357727

[B38] Rozas J. Ferrer-Mata A. Sánchez-DelBarrio J. C. Guirao-Rico S. Librado P. Ramos-Onsins S. E.. (2017). DnaSP 6: DNA sequence polymorphism analysis of large data sets. Mol. Biol. Evol. 34 (12), 3299–3302. doi: 10.1093/molbev/msx248 29029172

[B39] Rudall P. (1994). Anatomy and systematics of iridaceae. Botanical J. Linn. Soc. 114 (1), 1–21. doi: 10.1111/j.1095-8339.1994.tb01920.x

[B40] Rudall P. (1995). Anatomy of the monocotyledons. VIII. iridaceae (Clarendon Press).

[B41] Rudall P. J. Manning J. C. Goldblatt P. (2003). Evolution of floral nectaries in iridaceae. Ann. Missouri Botanical Garden 613-631. doi: 10.2307/3298546

[B42] Sharp P. M. Li W.-H. (1986). An evolutionary perspective on synonymous codon usage in unicellular organisms. J. Mol. Evol. 24 (1), 28–38. doi: 10.1007/BF02099948 3104616

[B43] Sinn B. T. Sedmak D. D. Kelly L. M. Freudenstein J. V. (2018). Total duplication of the small single copy region in the angiosperm plastome: rearrangement and inverted repeat instability in asarum. Am. J. Bot. 105 (1), 71–84. doi: 10.1002/ajb2.1001 29532923

[B44] Souza-Chies T. T. Bittar G. Nadot S. Carter L. Besin E. Lejeune B. (1997). Phylogenetic analysis ofIridaceae with parsimony and distance methods using the plastid generps4. Plant Systematics Evol. 204 (1), 109–123. doi: 10.1007/BF00982535

[B45] Sugiura M. (1992). “The chloroplast genome,” in 10 years plant molecular biology. Eds. Schilperoort R. A. Dure L. (Dordrecht: Springer). doi: 10.1007/978-94-011-2656-4_10

[B46] Tamura K. Stecher G. Peterson D. Filipski A. Kumar S. (2013). MEGA6: molecular evolutionary genetics analysis version 6.0. Mol. Biol. Evol. 30 (12), 2725–2729. doi: 10.1093/molbev/mst197 24132122 PMC3840312

[B47] Tang Y. Luo S. Zhang Y. Zhao Y. Li R. Guo L.. (2022). Comparative analysis about the structure of complete chloroplast genomes in genus mangifera and accuracy verification about phylogenetic analysis based on gene ycf2 in genus level. bioRxiv. doi: 10.1101/2022.04.05.487216

[B48] Tillich H.-J. (2003). Seedling morphology in iridaceae: indications for relationships within the family and to related families. Flora-Morphology Distribution Funct. Ecol. Plants 198 (3), 220–242. doi: 10.1078/0367-2530-00094

[B49] Tillich M. Lehwark P. Pellizzer T. Ulbricht-Jones E. S. Fischer A. Bock R.. (2017). GeSeq–versatile and accurate annotation of organelle genomes. Nucleic Acids Res. 45 (W1), W6–W11. doi: 10.1093/nar/gkx391 28486635 PMC5570176

[B50] Trifinopoulos J. Nguyen L.-T. von Haeseler A. Minh B. Q. (2016). W-IQ-TREE: a fast online phylogenetic tool for maximum likelihood analysis. Nucleic Acids Res. 44 (W1), W232–W235. doi: 10.1093/nar/gkw256 27084950 PMC4987875

[B51] van der Burgt A. Karimi Jashni M. Bahkali A. H. de Wit P. J. (2014). Pseudogenization in pathogenic fungi with different host plants and lifestyles might reflect their evolutionary past. Mol. Plant Pathol. 15 (2), 133–144. doi: 10.1111/mpp.12072 24393451 PMC6638865

[B52] Wakasugi T. Tsudzuki J. Ito S. Nakashima K. Tsudzuki T. Sugiura M. (1994). Loss of all ndh genes as determined by sequencing the entire chloroplast genome of the black pine pinus thunbergii. Proc. Natl. Acad. Sci. 91 (21), 9794–9798. doi: 10.1073/pnas.91.21.9794 7937893 PMC44903

[B53] Walker J. F. Zanis M. J. Emery N. C. (2014). Comparative analysis of complete chloroplast genome sequence and inversion variation in lasthenia burkei (Madieae, asteraceae). Am. J. Bot. 101 (4), 722–729. doi: 10.3732/ajb.1400049 24699541

[B54] Wang R.-J. Cheng C.-L. Chang C.-C. Wu C.-L. Su T.-M. Chaw S.-M. (2008). Dynamics and evolution of the inverted repeat-large single copy junctions in the chloroplast genomes of monocots. BMC evolutionary Biol. 8 (1), 1–14. doi: 10.1186/1471-2148-8-36 PMC2275221 18237435

[B55] Wang Y.-H. Wicke S. Wang H. Jin J.-J. Chen S.-Y. Zhang S.-D.. (2018). Plastid genome evolution in the early-diverging legume subfamily cercidoideae (Fabaceae). Front. Plant Sci. 138. doi: 10.3389/fpls.2018.00138 PMC5812350 29479365

[B56] Wicke S. Naumann J. (2018). “Molecular evolution of plastid genomes in parasitic flowering plants,” in Advances in botanical research (Elsevier), 315–347.

[B57] Wilgenbusch J. C. Swofford D. (2003). Inferring evolutionary trees with PAUP. Curr. Protoc. Bioinf. 1), 6.4. doi: 10.1002/0471250953.bi0604s00 18428704

[B58] Wu Z. Liao R. Yang T. Dong X. Lan D. Qin R.. (2020). Analysis of six chloroplast genomes provides insight into the evolution of chrysosplenium (Saxifragaceae). BMC Genomics 21 (1), 1–14. doi: 10.1186/s12864-020-07045-4 PMC7488271 32912155

[B59] Xia X. Xie Z. (2001). DAMBE: software package for data analysis in molecular biology and evolution. J. heredity 92 (4), 371–373. doi: 10.1093/jhered/92.4.371 11535656

[B60] Xu X. Wang D. (2021). Comparative chloroplast genomics of corydalis species (Papaveraceae): evolutionary perspectives on their unusual large scale rearrangements. Front. Plant Sci. 11, 600354. doi: 10.3389/fpls.2020.600354 33584746 PMC7873532

[B61] Yan M. Fritsch P. W. Moore M. J. Feng T. Meng A. Yang J.. (2018). Plastid phylogenomics resolves infrafamilial relationships of the styracaceae and sheds light on the backbone relationships of the ericales. Mol. Phylogenet. Evol. 121, 198–211. doi: 10.1016/j.ympev.2018.01.004 29360618

[B62] Yang Z. (2007). PAML 4: phylogenetic analysis by maximum likelihood. Mol. Biol. Evol. 24 (8), 1586–1591. doi: 10.1093/molbev/msm088 17483113

[B63] Zhong Q. Yang S. Sun X. Wang L. Li Y. (2019). The complete chloroplast genome of the Jerusalem artichoke (Helianthus tuberosus l.) and an adaptive evolutionary analysis of the ycf2 gene. PeerJ 7, e7596. 31531272 10.7717/peerj.7596 PMC6718157

[B64] Zhu A. Guo W. Gupta S. Fan W. Mower J. P. (2016). Evolutionary dynamics of the plastid inverted repeat: the effects of expansion, contraction, and loss on substitution rates. New Phytol. 209 (4), 1747–1756. doi: 10.1111/nph.13743 26574731

[B65] Zong D. Zhou A. Zhang Y. Zou X. Li D. Duan A.. (2019). Characterization of the complete chloroplast genomes of five populus species from the western sichuan plateau, southwest China: comparative and phylogenetic analyses. PeerJ 7, e6386. doi: 10.7717/peerj.6386 30809432 PMC6387583

