# Supplementary figures and images for: A phylogenomic study of Iridaceae Juss. based on complete plastid genome sequences
Source: Front Plant Sci. 2023 Jan 31;14:1066708. doi: 10.3389/fpls.2023.1066708 (PMC9948625; doi:10.3389/fpls.2023.1066708)

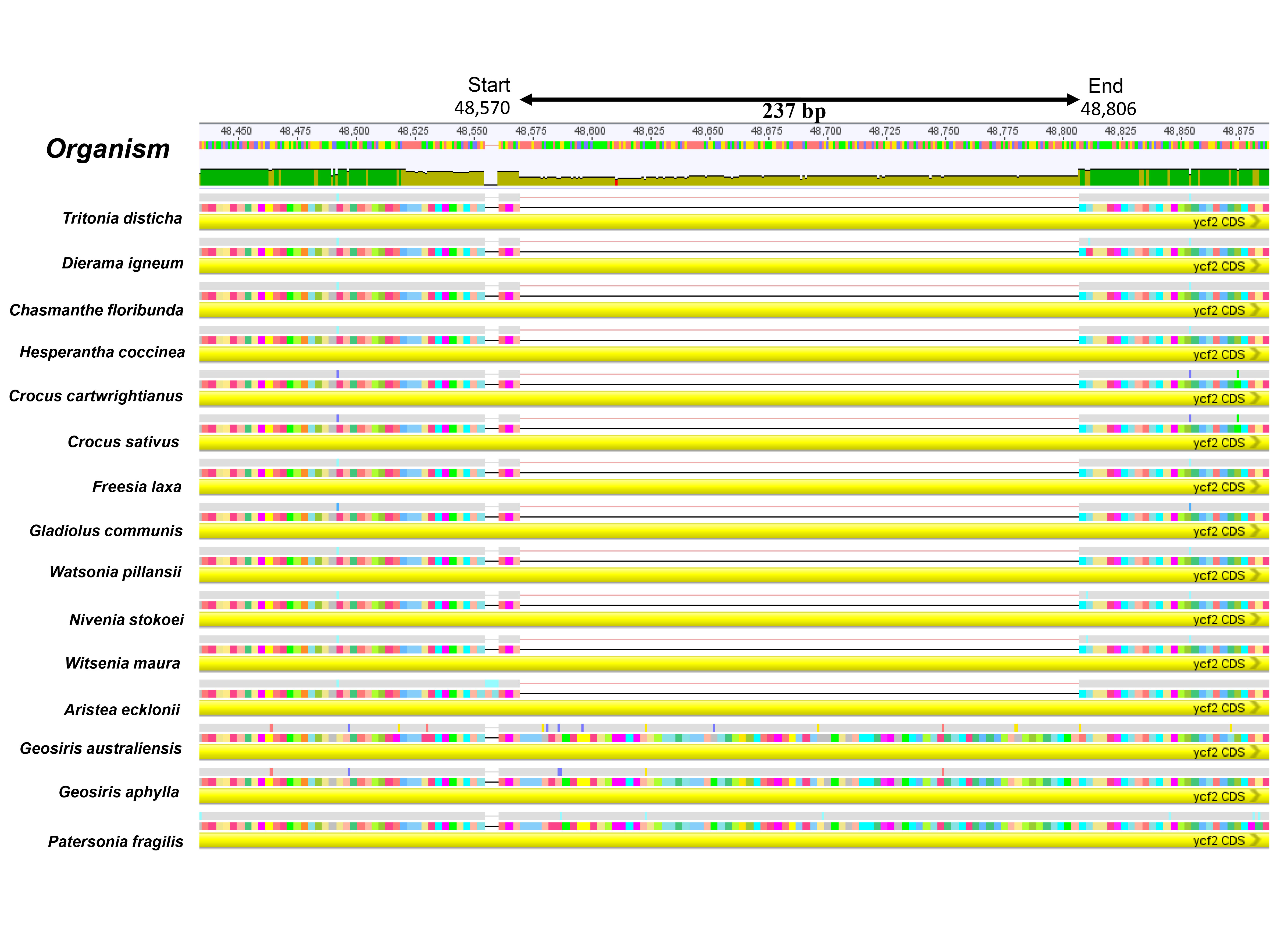

Supplement: Supplementary Figure 1 — Gene locus deletion in ycf2 gene shared by the sampled species of three subfamilies—Crocoideae, Nivenioideae and Aristeoideae—of Iridaceae. The white gap corresponds to the missing amino acid sequence. [file Image_1.jpeg]

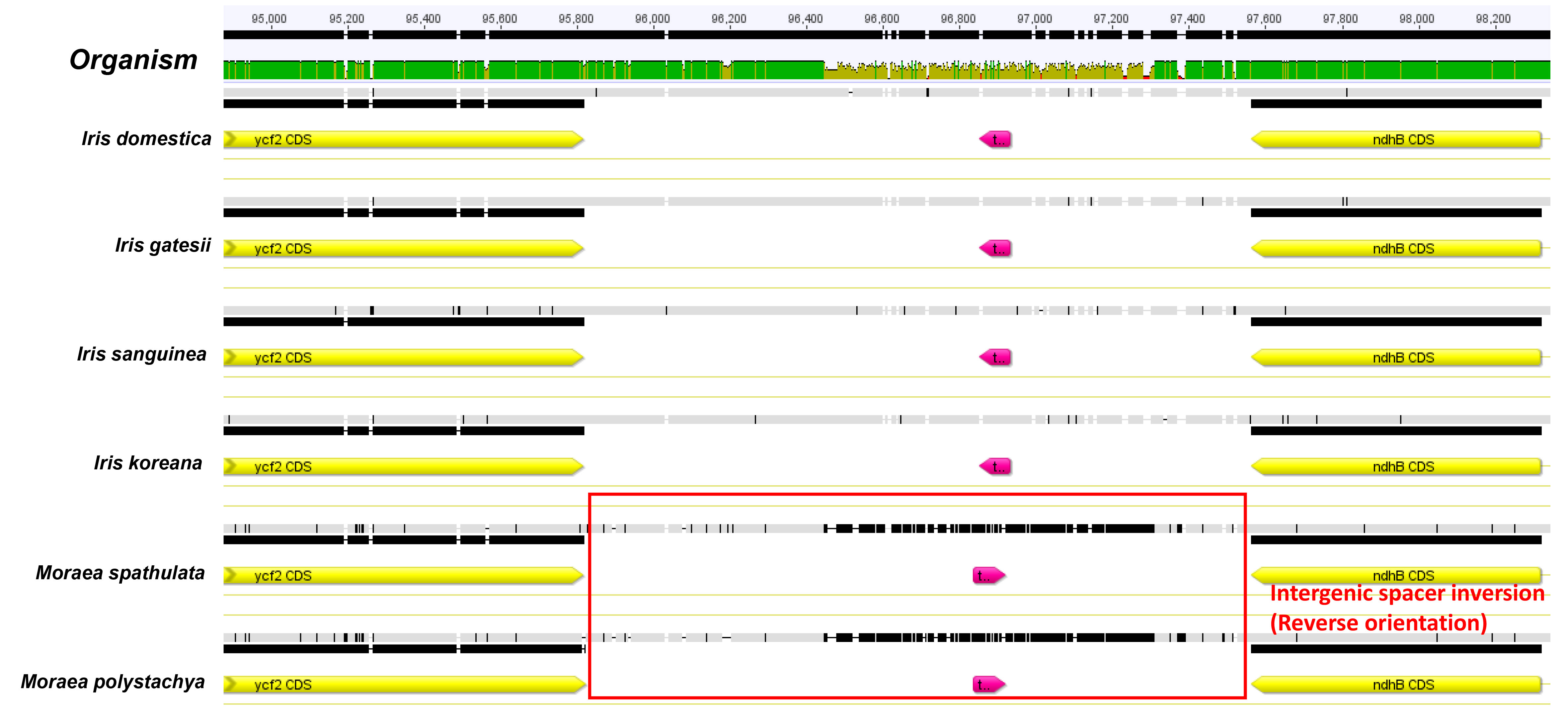

Supplement: Supplementary Figure 2 — Inversion events. [file Image_2.jpeg]

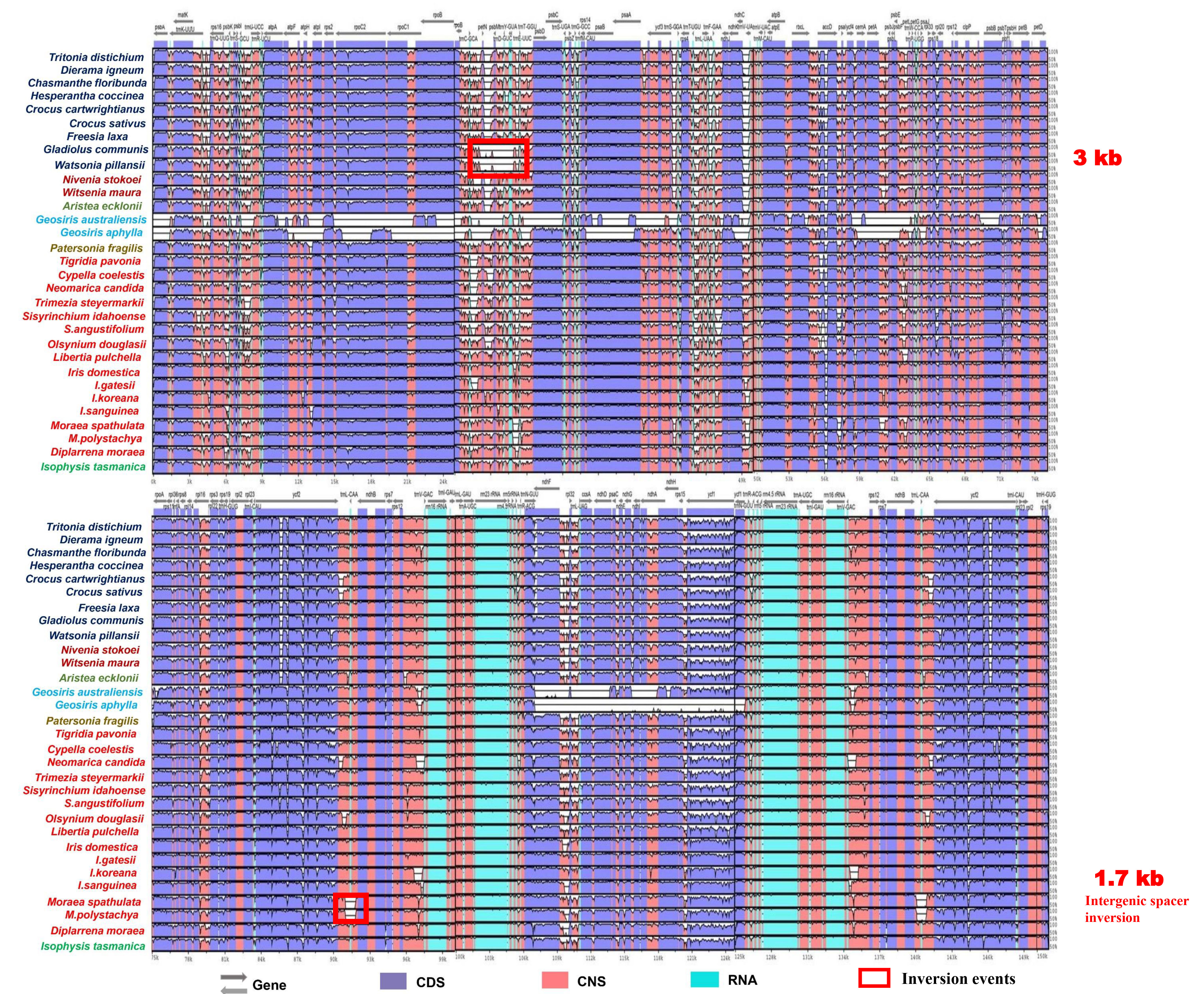

Supplement: Supplementary Figure 3 — Inversion events. [file Image_3.jpeg]

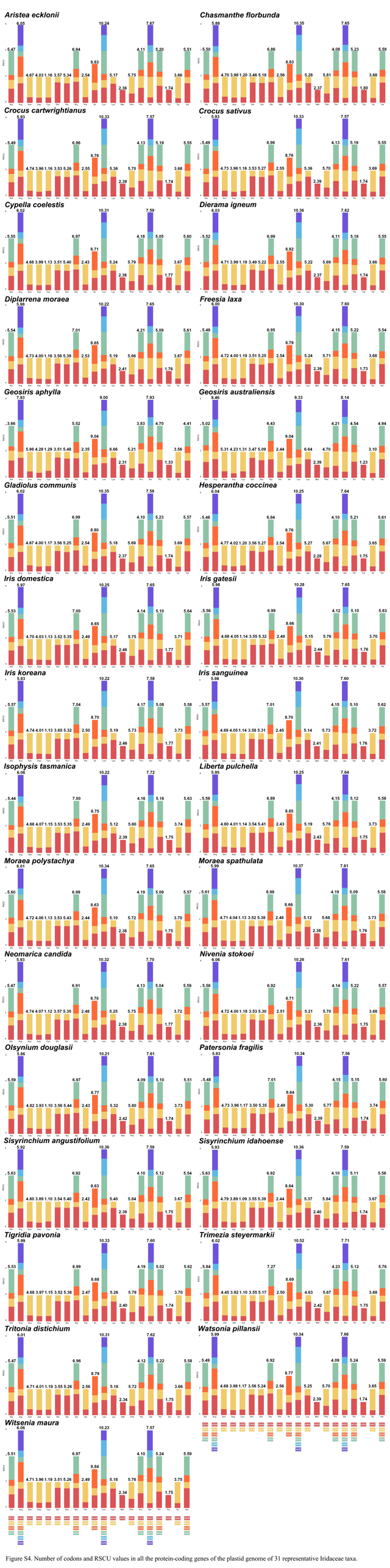

Supplement: Supplementary Figure 4 — Number of codons and relative synonymous codon usage values in all the protein-coding genes of the plastid genome of 31 representative Iridaceae taxa. [file Image_4.jpeg]
